# Validation and normative data for a new dynamic instrument evaluating phonological processing skills in Italian first-grade students

**DOI:** 10.3389/fpsyg.2025.1655268

**Published:** 2026-01-07

**Authors:** Silvia Stefanelli, Giorgia Zaghini, Erika Benassi, Francesca Vedovati, Manuela Gragnaniello, Enrico Savelli, Giacomo Stella

**Affiliations:** 1 Department of Humanities, University of the Republic of San Marino, San Marino, San Marino; 2 Department of Education and Humanities, University of Modena and Reggio Emilia, Reggio Emilia, Italy; 3 Azienda Sanitaria Locale Roma 4 TSMREE Distretto 2, Ladispoli, Roma, Italy

**Keywords:** Developmental Language Disorder (DLD), dynamic assessment (DA), early identification, first-grade students, Italian-speaking children, phonological processing abilities, Speech Sound Disorder (SSD)

## Abstract

**Introduction:**

Phonological processing skills are fundamental to children's language development and academic success. There is an increasing need for assessment tools that can reliably identify difficulties in these areas at the beginning of primary school. This concerns both children already diagnosed with Developmental Language Disorder (DLD) or Speech Sound Disorder (SSD) and those without a diagnosis who may still have a weak phonological system.

**Aim:**

This study introduces a new tool for assessing phonological processing skills, called *Parla-dino test*, based on a dynamic assessment approach. The tool allows the observation of modifiability in the child's productions through graduated prompts, integrating quantitative performance with qualitative information.

**Materials and method:**

A sample of 105 Italian monolingual first-grade children was administered both the Parla-dino and Cossu's Phonological Processing Test (gold standard measure) to assess the convergent validity. Additionally, 15 first-grade children with DLD were compared to a matched group of 45 typically developing (TD) peers to examine discriminant validity.

**Results:**

Significant correlations were found between the Phonological Processing Test and both the static and dynamic scores on Parla-dino test (r_s_=0.74, *p* < 0.001; r_s_=0.78, *p* < 0.001). Children with DLD performed significantly worse than their TD peers on both static (U = 101, *p* < 0.001) and dynamic scores (U = 124, *p* < 0.001). Logistic regression showed that the dynamic score emerged as a significant predictor of group status (B = −0.128, SE = 0.037, Z = −3.39, *p* < 0.001), indicating that higher *Parla-dino* scores were associated with a lower likelihood of being classified in the DLD group. Specificity and sensitivity were calculated, and normative data for Italian first-grade students were provided.

**Conclusions:**

Children with DLD who exhibit specific impairments in phonological processing showed limited modifiability in word production, even when facilitative cues were provided. This behavioral pattern may represent a potential marker of phonological processing difficulties and can contribute to a better understanding of DLD phenotypes. Accordingly, the *Parla-dino* test appears to be a valid and effective tool for identifying phonological vulnerability in Italian children at the beginning of primary school.

## 1 Introduction

Language development appears compromised in approximately 5–7% of children over 3 years old (Law et al., 2000; Wallace et al., 2015), and Developmental Language Disorder (DLD) is one of the most prevalent neurodevelopmental disorder in childhood (Iverson and Williams, 2025; Rapin, 2006). At the time of starting school, around two children in every class of 30 have a language disorder that is severe enough to impede their academic progress (Norbury et al., 2016). In Italy, the prevalence of DLD has been estimated to be 11.7% at 4–5 years of age, declining to around 4% by the time children enter primary school (CLASTA and FLI, 2019; Rinaldi et al., 2023).

DLD is characterized by “*persistent difficulties in the acquisition and use of language across modalities (i.e., spoken, written, sign language, or other) due to deficits in comprehension and production without cognitive, medical, or neurological conditions, (…) with the onset in the early developmental period*” (DSM-5-TR, American Psychiatric Association, 2022, p.48).

One critical aspect of language development is the ability to produce speech sounds accurately in words. A specific impairment in this domain, known as Speech Sound Disorder (SSD) or Phonological Disorder (DSM-5-TR, American Psychiatric Association, 2022), affects the articulation of phonemes (i.e., individual sounds) and the ability to combine phonemes in words. In observing language development among typically developing (TD) Italian children, the phonetic inventory usually completes by age three, although some residual difficulties, such as rhotacism and sigmatism, may persist (Bortolini, 2004; Stella, 2013). The child's acquisition of individual phonemic units does not guarantee accurate word production because, in spoken language, sounds are produced not in isolation but are coarticulated with precise timing and coordinated articulatory movements (Archibald and Joanisse, 2012; Michelazzo and Eramo, 2009; Pinton et al., 2014). The production of each phoneme in a word is influenced by the sounds that precede and follow it. Thus, the correct production of words requires both phonetic knowledge of the sounds, phonological representation abilities, and motor planning and coordination of the movements of the articulators (such as the jaw, tongue, and lips) (DSM-5 TR, APA, 2022). All these processes, referred to as “phonological processing”, seem crucial for language and literacy acquisition (Archibald and Joanisse, 2012; Connine and Darnieder, 2009; Melby-Lervåg et al., 2012; Michelazzo and Eramo, 2009; Wagner and Torgesen, 1987).

Children with SSD may have persistent difficulties producing speech sounds, which can include adding, omitting, distorting, or substituting phonemes (ASHA American Speech-Language-Hearing Association, 2014). These issues can impact speech intelligibility, communication, peer interaction, and academic achievement (DSM-5 TR, APA, 2022). These children may also struggle with perceiving sound contrasts (Archibald and Joanisse, 2012; Connine and Darnieder, 2009; Tallal et al., 1985), show a significantly slower articulation rate (measured as syllables per second) compared to their peers (Peter, 2012), and encounter difficulties in organizing complex syllables. Additionally, they face challenges in resolving phonological processes, such as weak syllable reduction, consonant or vowel deletion, consonant harmony, and cluster simplification, which persist beyond typical developmental stages (Pinton et al., 2014; Stella, 2013; Zanobini et al., 2012). When supported with speech therapy, these children generally improve, demonstrating enhanced speech intelligibility and the acquisition of more complex syllabic structures (Scarborough and Dobrich, 1990; Rinaldi et al., 2021). However, the disorder can follow different trajectories and persist into later ages (DSM-5 TR, APA, 2022). At the beginning of primary school, children with SSD may still struggle to produce low-frequency and phonotactically complex words, as well as with phonological awareness (Pinton et al., 2014). These difficulties can significantly impact the acquisition of literacy skills (Angelelli et al., 2016; Brizzolara et al., 1999; Ramus et al., 2003), thereby increasing the risk of Learning Disabilities (Bishop and Adams, 1992; Cantiani et al., 2015; Chilosi et al., 2009; Conti-Ramsden et al., 2012; Durkin et al., 2013; Yaşa and Çiyiltepe, 2024). The phonological deficit theory, one of the main explanatory theories of developmental dyslexia and spelling disorders (Ramus et al., 2013), posits that the literacy challenges of these children stem from a linguistic deficit specifically related to the representation and processing of speech sounds (Snowling, 2000). Consequently, assessing phonological processing abilities at the end of kindergarten and the first year of primary school in children with linguistic difficulties could be crucial for identifying risk factors and initiating timely interventions (De Cagno and Ceccarelli, 2013). Through clinical assessment of phonological processing, potential challenges in spelling and difficulties accessing lexical resources, as well as integrative processes and specialized lexicons characterized by complex morphology, can be anticipated (Stella, 2013).

Phonological processing is typically assessed using word repetition tasks involving low-frequency and phonotactically complex stimuli. This type of assessment is static, meaning it evaluates the child's current level of performance by measuring the presence or absence of phonemes and identifying phonological processes used when repeating real (and also nonwords) of increasing complexity (Camilleri and Law, 2007; Elliott, 2003; Haywood and Tzuriel, 2002). Repetition of real words engages the phonological form of lexical representations stored in long-term memory, which reflect not only phonological but also semantic knowledge. The influence of long-term lexical knowledge on temporary verbal storage is evidenced by higher accuracy in repeating real words compared to nonwords in Italian-speaking children (Dispaldro et al., 2009). In Italy, the availability of standardized clinical tools for assessing phonological processing is limited. One widely used static diagnostic task, developed by Cossu (n.d.), involves the repetition of complex low-frequency words and a small number of nonwords. It is typically administered to children in primary school. Nonword repetition tasks are included in several assessment tools in Italy, such as the *Battery for the Assessment of Language in Children 4–12* (Marini et al., 2015), which incorporates a nonword repetition subtest to evaluate phonological processing. However, as noted by (Gathercole 2006), performance on nonword repetition tasks is strongly influenced by phonological short-term memory, which plays a significant role in task execution. Some studies (Archibald, 2008; Coady and Evans, 2008; Piazzalunga et al., 2019) suggest that this type of test primarily measures phonological short-term memory, although involves multiple linguistic processes, such as language perception, phonological encoding, motor planning, and articulation. Although standardized tests are validated and reliable, their controlled administration settings may limit ecological validity and contextual applicability (Glaspey and Stoel-Gammon, 2007). For this reason, integrating standardized measures with qualitative methods can provide a richer and more realistic picture of children's phonological and linguistic competence. For example, some clinicians assess phonological skills by recording and coding the child's spontaneous speech (Gherardi et al., 2006). Nevertheless, this latter approach is not always feasible due to time constraints, and it does not always reveal phonological difficulties in school-aged children.

Dynamic assessment (DA) represents an alternative or a supplemental approach to traditional language assessments. It is grounded in Vygotsky's socio-cultural theory (Vygotsky, 1978; Bain and Olswang, 1995), which distinguishes between a child's actual developmental level—what the child can do independently—and potential developmental level—what the child can achieve with guidance. The gap between these levels defines the “Zone of Proximal Development” (ZPD; Vygotsky, 1978), emphasizing both the learning process and the learning outcome. Within this framework, DA examines learning through child-examiner interaction, providing tailored and gradual supports (Gutiérrez-Clellen and Peña, 2001) to identify the ZPD. Unlike traditional tests, DA is interactive and adaptive, focusing on a child's ability to improve with support rather than merely comparing performance to peers (Tzuriel, 2012; 2021). A key distinction exists between research-oriented and clinical models of DA (Tzuriel, 2021). The research-oriented model, typically referred to as the test–teach–retest model, is used to quantify learning potential and cognitive modifiability under controlled conditions. The clinical model, instead, emphasizes the mediated learning experience as an interactive diagnostic–therapeutic process, where the examiner flexibly adapts prompts and feedback to the child's needs and uses the results to inform individualized intervention planning. Two main methodological formats are commonly used within DA. The test-teach-retest approach method involves an initial static assessment, followed by a phase where the clinician teaches the task principles through interactions that utilize mediated learning experience with the child and concludes with a reassessment to measure skill modifiability after the intervention (Peña et al., 2014; Petersen et al., 2017). The graduated prompting method involves providing the child with a predetermined hierarchical series of prompts until the linguistic goal is achieved (Gutiérrez-Clellen and Peña, 2001). Graduated prompting is often accompanied by a modifiability scale, where the clinician rates the child on a Likert scale based on attention, motivation, self-regulation, and the intensity of effort required to induce change (Kehoe and Matrat, 2024). According to (Tzuriel 2021), improvements observed during DA should be interpreted as indicators of potential change rather than as evidence of stable acquisition. Long-term and permanent modifications in underlying skills—such as those related to phonological processing—are likely to occur only when the insights gained through DA are followed by intensive and systematic intervention designed to consolidate the learning mechanisms activated during assessment. In phonology testing, the DA is less developed compared to other language domains; there are some dynamic tasks in English, such as the Glaspey Dynamic Assessment of Phonology (Glaspey, 2019) and Dynamic Evaluation of Motor Speech Skill (Strand and McCauley, 2013). The Glaspey assessment is designed for children aged 3 to 10 years and evaluates the production of English phonemes and some consonant clusters across different linguistic contexts (isolation, word, short sentence, and connected speech). It includes four levels of cueing: spontaneous production without support, verbal instruction or model, production with prolongation or segmentation, and production with tactile prompts. The assessment developed by (Strand and McCauley 2013) is intended for children with limited phonetic inventories. It evaluates speech production by providing visual, temporal, and tactile supports to help the child produce target words. Both tests feature a dynamic scoring system where a lower score indicates the child can produce the word independently (without instructions or verbal prompts), while a higher score indicates reliance on multiple support strategies. Additionally, (Hasson et al. 2013) included a dynamic phonological assessment using a test-teach-retest approach as part of a battery for assessing language skills in bilingual children. In this assessment, the child names 10 pictures; if the sounds are not produced correctly, the clinician models the correct pronunciation, prompting the child to imitate it to assess stimulability. Subsequently, the child renames all 10 pictures. A comparison between the initial and final production determines the effectiveness of sound instruction in improving word pronunciation. (De Marchi et al. 2003) developed the Evaluation Dynamique de la Phonologie, a test for French children aged 3–6 years, which assesses phonetic-phonological expressive levels in different production contexts, from connected speech to single words.

Currently, dynamic assessments for evaluating children's phonological skills are lacking in Italy. The present study aimed to verify the convergent and discriminant validity of a new DA tool, called *Parla-dino*, designed to evaluate the child's phonological processing skills using complex word structures and graduated prompts. Due to its characteristics, this tool may prove effective in identifying children with phonological difficulties or with a phonological system that is not yet fully stabilized in first-grade schoolers. *Parla-dino* may offer an assessment approach that takes into account children's “modifiability”, enabling timely interventions to reduce psychological and adaptive challenges and to prevent future learning difficulties. The study aimed to provide normative data for Italian first-grade students.

## 2 Materials and methods

### 2.1 Participants

The study included 105 typically developing first graders (age in months, mean = 80.1, SD=3.63, range 72–86; 59 males, 46 females), recruited from five Italian primary schools. Participants can be considered monolingual: children whose first language was not Italian (*n* = 19) and those with two foreign parents (*n* = 10) were excluded from the sample. Students had non-verbal intelligence within the normal range: we excluded participants with ≤ 20th percentile at Raven Coloured Progressive Matrices (CPM; Italian adaptation by Belacchi et al., 2008). Seven students with Neurodevelopmental Disorders completed the tasks, but their data were also excluded from the data analysis (e.g., intellectual disability; DLD with/without attention-deficit/hyperactivity disorder; autism spectrum disorder). Additionally, data from 7 children who exhibited rhotacism were excluded (see measures section for details). At the time of data collection, approximately 3% (*n* = 3) of the sample were receiving speech-language or psychological therapy, and 12% (*n* = *13*) had previously received such interventions. Regarding parental education, 31% of mothers (*n* = 33) had completed high school or less (including lower secondary and upper secondary education), while 69% (*n* = 72) held a university degree or higher (including undergraduate and postgraduate degrees). In contrast, 63% of fathers (*n* = 65) had completed high school or less, and 37% (*n* = 38) held a university degree or higher.

The study also included a clinical group of 15 first-grade children with DLD (DLD group; age in months, mean = 79.3, SD=3.37, range 74–85; 11 males, 4 females), recruited from private centers specializing in the diagnosis and intervention of language and learning disorders. All children were born in Italy and had non-verbal intelligence within the normal range. Within this group, only two children had one non-Italian parent, and one child had two foreign parents. Each child had a diagnosis of DLD according to DSM-5 TR criteria (APA, 2022), and 13 (87%) had either previously received or were currently receiving speech-language therapy.

According to the World Medical Association Declaration of Helsinki's ethical principles, informed consent was obtained from the parents or legal guardians of the students, and children gave verbal consent to participate in the study. The study was approved by the Department of Humanities of the University of San Marino (prot. num. 601, 15/02/2022).

### 2.2 Measures

The phonological processing skills were examined using two instruments: (1) the task elaborated by Cossu (n.d.), widely used as a diagnostic test for identifying phonological processing difficulties in Italian school-age children; (2) the new dynamic test developed by the authors and called *Parla-dino*.

#### 2.2.1 Phonological Processing Test (Test di Programmazione Fonologica)

Phonological Processing Test (Cossu, n.d.) consists of 31 three-syllable, four-syllable, five-syllable, and six-syllable words (e.g., “diverbio”, “chiedibile”, “termosifone”, “insostenibile”) and some non-word (e.g., “duvacine”, “tendometro”). All real words are of medium-low frequency, cover all Italian consonants and vowels, and vary across typical Italian phonemic contrasts. The child is asked to repeat the word after the examiner pronounces it at normal pitch and loudness. The number of phonological errors and the number of words correctly repeated (total raw score) were recorded by the examiner. If a child self-corrects spontaneously, the error is not counted. For each target word, we calculated the number of phonological processes that the child produced. Each phonological process is assigned one point. If multiple processes occur in a word, the total number of processes is summed (e.g.,/di'vεrbjo/realized as/di'vεbo/, with two processes identified—consonant cluster reduction and diphthong reduction—resulting in a score of 2). However, if a child produces three or more phonological processes in a single word, the maximum score assigned remains at 3. Additionally, idiosyncratic productions, failure to repeat the word, or only partial repetitions (e.g., repeating only one syllable) are also scored with a maximum of 3 points.

#### 2.2.2 Parla-dino: a new dynamic Phonological Processing Test

*Parla-dino* was developed based on a preliminary empirical study, initially designed by Giacomo Stella and conducted as a student research project by Uccello and Fontana (2017–2018 academic year, *unpublished*). Words for the *Parla-dino* task were selected from the Italian children's lexicon collected by (Marconi et al. 1994), which provides a list of commonly used words among primary school children in both reading and writing. The words (nouns and adjectives) are chosen based on three criteria: word length, frequency, and morphological structure. The selected words are characterized by low frequency to reduce the effects of lexical knowledge and retrieval on speech production. These words are also multisyllabic to stress the co-articulation process and can be categorized into compound and derived words to guarantee the manipulation of their morphological structure.

P*arla-dino* is a phonological processing task involving the repetition of 15 words (5 compound and 10 derived words). The child is instructed to merely repeat, as accurately as possible, the words spoken by the examiner. If a child struggles to produce the initial target word, the examiner supports him with graduated prompts that simplify the phonological complexity of the word. The mediation provided in *Parla-dino* is strictly linguistic and limited to morphological and phonological simplifications (e.g., *ferroviario* progressively simplified as *ferrovia* and then *ferro*); no metacognitive, articulatory, or explicit teaching strategies are introduced This DA aims to determine the current level of phonological processing and the ZPD (Vygotsky, 1978).

*Parla-dino* assesses the child's performance through two distinct parameters: static and dynamic score. The static score ranges from 0 to 15 and is based on the accuracy of the target word during the first repetition. One point is given for each correctly repeated word on the first attempt. If a child self-corrects spontaneously, the error is not counted. The dynamic score ranges from 0 to 60, and it measures the child's skill in repeating the target word with the provided prompt (i.e., linguistic simplification). Morphological components are thus simplified using graduated prompts, moving toward the simplest thematic structure, which retains some phonotactic characteristics of the initial word but is shorter in length. The use of morphologically simple words allows for the evaluation of the child's ability to produce complex articulatory elements within shorter phonological structures. This method helps identify whether difficulties in repeating complex words stem from morphological complexity, and thus increased articulatory demands, or from the length of the articulatory plan. Points are awarded as follows:

- 4 points for a correct response on the first attempt;
- 3 points if the child correctly repeats the first simplification and then correctly says the entire target word;
- 2 points if the first simplification is correct, but the complete target word is not;
- 1 point is awarded in the case of compound words if only one part of the compound is repeated. For derived words, it is given if only the second simplified cue is correctly produced.
- 0 points if none of the repetitions are correct.

Specific scoring guidelines were applied to compound and derived words (for detailed examples, see Table 1):

- For compound words, the first prompt involves the separate repetition of the two constituent words. If the child correctly produces both parts and the entire word is correctly repeated, the score is 3 points. If the child correctly produces only the two parts but not the entire compound word, the score is 2 points. If only one part is correctly produced, 1 point is awarded; otherwise, 0 points are given.
- For derived words, the first prompt involves the base word from which the target word is derived. If the child correctly produces this base word and then repeats the entire derived word correctly, 3 points are awarded. If the derived word is incorrect, 2 points are given. If the initial simplification is still incorrect, the second cue involves presenting the simplest form of the initial word. If this simple form is correct, 1 point is awarded; otherwise, 0 points are given.

**Table 1:** Scoring examples for compound and derived words.

**Type**	**Examples**	**Points**				
		**4**	**3**	**2**	**1**	**0**
Compound	/altopar'lante/ [loudspeaker]	Correct repetition of/altopar'lante/	/'alto/and /par'lante/ are correctly repeated as well as the second repetition of/altopar'lante/	/'alto/and/par'lante/ are correctly repeated, but the second repetition of/altopar'lante/is not.	/'alto/ is correctly repeated but/par'lante/is incorrect.	/'alto/and/par'lante/ are incorrect.
Derived	/fer:o'vjarjo/ [railway]	Correct repetition of/fer:o'vjarjo/	Correct repetition of/fer:o'vja/and second repetition of/fer:o'vjarjo/	/fer:ovja/ is correctly produced, but the second repetition of/fer:o'vjarjo/is incorrect.	/fer:o'vja/ is incorrect but/'fer:o/is correct.	/'fer:o/ is incorrect.

For a qualitative assessment, *Parla-dino* also allows for the analysis of the phonological processes observed during a child's word production. Importantly, when a phoneme is absent from the child's phonetic inventory, phonological analysis of that phoneme is not applicable. For example, in the case of the phoneme/r/, if it is either absent or consistently realized with articulatory distortion (i.e., rhotacism), this phenomenon is documented as a specific articulatory deficit rather than a phonological process in the strict sense. Similarly, phonetic-phonological deviations attributable to dialectal variation, phonetic inaccuracies, or articulatory-based alterations —such as inaccurate realizations of individual phonemes—should not be interpreted as phonological errors (e.g.,/medjamente/may be produced with either an open [ε] or close [e] vowel). These include but are not limited to, interdental or lateral sigmatism (articulatory distortions of/s/that do not compromise intelligibility), distinctions between open and closed vowels, consonant gemination, vowel lengthening, and the production of/ts/with the tongue interdentally. The phonological analysis is conducted in terms of phonological processes, following the classification proposed by (Bortolini 2004; for the translation of the name of the processes in Italian, see Gironda and Fabus, 2011), which distinguishes between structural (syntagmatic relationships between phonological units) and systemic processes (accurate perception but difficulty in contrastive use of sounds). The test also considers the process of substituting consonants or vowels, where one sound is replaced by another that doesn't fit into the previously described processes. Additionally, “unusual processes” are considered—these involve atypical simplifications in speech development, such as leaving out sounds in clusters and weakening of stops. Words that exhibit three or more phonological processes are classified as idiosyncratic productions.

After phonetic transcription of the child's productions, each incorrectly produced word is analyzed to identify the specific phonological processes carried out by the child. Each identified process is assigned one point. If multiple processes occur in a single word, the total number of processes is summed. However, a maximum of 3 points is assigned per word even if more than three processes are present. Similarly, idiosyncratic productions, failure to repeat the word, or partial repetitions (e.g., repeating only one syllable) are also scored with a maximum of 3 points. The total phonological process score ranges from 0 to 45 points.

The *Parla-dino* test, including detailed administration guidelines, scoring manuals, examples of specific graduated prompts (phonological and morphological simplifications for each target word), and a short tutorial video, will be freely available for download from the official website of the Department of Humanities of the University of San Marino to ensure procedural consistency and examiner reliability.

#### 2.2.3 Colored Progressive Matrices

The CPM (Raven et al., 1998; Italian adaptation by Belacchi et al., 2008) is an individually administered, non-verbal intelligence test that measures reasoning and visual–spatial problem-solving abilities. The task consists of selecting the missing element from a matrix of geometric figures. Raw scores were converted into age-referenced normative values based on the Italian standardization.

### 2.3 Procedures

A speech-language therapist conducted data collection for TD children between February and March 2024. To minimize examiner-related variability as much as possible, all sessions in this study were conducted by the same experienced speech-language therapist to ensure consistency in administration. Before data collection, parents completed a questionnaire to gather sociodemographic information (e.g., nationalities of the child and parents, parents' education levels) and personal details (e.g., child's first language, history of DLD, previous or ongoing speech therapy). The socioeconomic status of the participants' families was determined using some indicators from the Italian translation of the (Hollingshead 1975) Four Factor Index of Social Status. All data were individually collected in a quiet classroom organized by the teachers. The CPM test (Belacchi et al., 2008) was used to assess nonverbal intelligence, followed by two tasks that evaluated phonological processing. Children's responses to the phonological tasks were recorded to ensure accurate phonetic transcription, which was completed after the experimental session using the International Phonetic Alphabet (IPA) system.

Data was collected for children with DLD between January and March of 2024 and 2025. The procedure was the same as that administered to typically developing children, but the sessions took place in a clinical setting.

Data were collected anonymously using alphanumeric codes, ensuring individual participants could not be identified. These data were stored in an electronic database.

### 2.4 Statistical analysis

The statistical analyses were run using The jamovi project (2024) and R Core Team (2024). As a first step, descriptive analyses of the TD sample revealed two outliers in the *Parla-dino* static and dynamic scores, which were identified as extreme values through boxplot analysis. These outliers were subsequently excluded from the sample. Then, the normality of *Parla-dino* score distributions was tested using the Shapiro–Wilk test (W = 0.93, *p* < 0.001). Non-parametric tests were applied due to the non-normal distribution of the data. Given the non-normality of the variables, percentiles were used instead of means and standard deviations to define performance thresholds on the *Parla-dino* test. Although cutoff criteria still vary across international literature, the threshold indicating a phonological processing deficit was set at the 10th percentile, a commonly used cutoff in diagnostic measures for identifying DLD (see CLASTA and FLI, 2019; Sansavini, 2021, p. 43).

The convergent validity of the *Parla-dino* test was assessed by correlating raw scores on the *Parla-dino* with those on the Phonological Processing Test. As both variables were expressed on comparable scales, with higher values indicating better performance, no data transformation was applied. Because the distributions deviated from normality, non-parametric Spearman's rho correlations were computed to assess the correlations between measures. We also computed the partial correlation (corrected by age) between the scores for the two tasks (Kim, 2015).

To examine the discriminant validity of the *Parla-dino*, comparisons were performed between 15 children with DLD and 45 individually matched typically developing peers (matched for age, gender, and non-verbal intellingence; Mann–Whitney U = 219, *p* =0.319). This subsample was used to control for potential confounding factors and to ensure balanced groups for the analysis of diagnostic utility. A detailed description of the sociodemographic characteristics of these two groups is presented in Table 2. Additionally, a binary logistic regression was conducted to explore the relationship between the dynamic *Parla-dino* score and group classification (DLD vs. TD). The model's discriminant accuracy was evaluated using the Area Under the Curve (AUC) from Receiver Operating Characteristic analysis (Sing et al., 2020). An AUC value above 0.8 is typically considered indicative of strong discriminative ability (Hosmer et al., 2013). In this analysis, the dynamic *Parla-dino* score was used as the predictor (independent variable), with group status (DLD vs. TD) as the outcome (dependent variable). Given the characteristics of the sample, we also fitted a Firth-penalized logistic regression model to reduce potential small-sample bias in maximum-likelihood estimates. This analysis was conducted in addition to the standard logistic regression. The sensitivity, defined as the proportion of individuals with the DLD correctly identified by the test, and specificity, the proportion of individuals without the disorder who are correctly classified, were also calculated. Sensitivity and specificity values of at least 80% are acceptable (Meisels, 1988).

**Table 2:** TD and DLD groups: sample sociodemographic characteristics (*n* = 60).

**Variables**	**TD group (*****n** =* **45)**		**DLD group (*****n** =* **15)**	
	***n***	**%**	***n***	**%**
**Gender**				
Female	12	27%	4	27%
Male	33	73%	11	73%
**Highest maternal education level**				
High school or less	18	40%	10	71%
University degree or higher	27	60%	4	29%
**Highest paternal education level**				
High school or less	30	68%	10	83%
University degree or higher	14	32%	2	17%
**Age in months**				
M (SD) range	80.9 (2.77) 75–85		79 (3.37) 74–85	
**CPM (raw score)**				
M (SD) range	22.4 (3.03) 16–28		21.6 (3.65) 16–29	

## 3 Results

### 3.1 Convergent and discriminant validity

The convergent validity of the *Parla-dino* test was assessed by comparing its scores with those of the Phonological Processing Test (Cossu, n.d.). The *Parla-dino* scores closely correlated with the scores on the Phonological Processing Test, providing evidence for its convergent validity. Specifically, we conducted Spearman's correlation analyses to examine the relationships between the number of phonological errors in the two tasks, revealing a significant, strong positive correlation (r_s_=0.72, *p* < 0.001). Additionally, there were significant associations between the total Phonological Processing Test score and the static and dynamic scores of *Parla-dino* (*r*_*s*_=0.74, *p* < 0.001; *r*_*s*_=0.78, *p* < 0.001). In other words, the higher the level of accuracy in the Phonological Processing Test, the higher the competencies in our task. The correlations remained significant even after controlling for age in months (see Table 3).

**Table 3:** Spearman's partial correlations (corrected for age) between the scores at *Parla-dino* test and the scores at the Phonological Processing Test (*n* = 105).

***Parla-dino***	**Phonological processing test**	
	**Total score (accuracy)**	**Phonological errors**
Static score (accuracy)	0.72^***^	-
Dynamic score (accuracy)	0.76^***^	-
Phonological errors	-	0.70^***^

^***^p < 0.001.

To investigate the discrimination of *Parla-dino* among a clinical cohort of children with DLD, we compared their outcomes to those of the TD control group (matched peers). Mann-Whitney analyses revealed significant differences between the groups: children with DLD performed significantly worse than their TD peers on both static (Mann–Whitney U = 101, *p* < 0.001) and dynamic scores (Mann–Whitney U = 124, *p* < 0.001). The number of phonological processes also differed significantly, with the DLD group producing approximately twice as many phonological errors as the TD group (Mann–Whitney U = 119, *p* < 0.001) (see Table 4).

**Table 4:** Results of the statistical comparisons between the children with DLD (*n* = 15) and TD matched peers (*n* = 45).

***Parla-dino***	**DLD group (** ***n** =**15*** **)**			**TD group (** ***n** = **45*** **)**			***p***
	***M***	***SD***	**Range**	***M***	***SD***	**range**	
Static raw score (accuracy)	5.73	3.06	0–11	10.29	3.13	2–15	< 0.001
Dynamic raw score (accuracy)	37.27	12.10	13–53	50.02	7.89	28–60	< 0.001
Phonological process (errors)	16	7.72	6–35	7.53	5.64	0–24	< 0.001

To support the discriminant validity of the *Parla-dino*, we conducted a binary logistic regression analysis to examine the extent to which the children's performance on the task predicted their group status (DLD vs. TD). The results revealed a statistically significant and robust association between the dynamic *Parla-dino* test score and group status. The model demonstrated good overall fit (McFadden's R^2^ = 0.245; χ^2^(1) = 16.5, *p* < 0.001) and a satisfactory ability to discriminate between groups, as indicated by an AUC of 0.817, reflecting good classification accuracy. Crucially, the dynamic score was a significant predictor of group status (B = −0.128, SE = 0.037, Z = −3.39, *p* < 0.001), indicating that higher scores on the *Parla-dino* test were associated with a lower probability of being classified in the DLD group. Specifically, for each one-point increase in the dynamic score, the odds of belonging to the DLD group decreased by approximately 12% (Odds Ratio = 0.880). These results indicate that the *Parla-dino* task effectively discriminates this clinical population from TD peers. To verify the robustness of the findings, we also performed a Firth-penalized logistic regression. The penalized estimates were virtually identical to those obtained with the standard model (B = −0.13, SE = 0.04), and the effect remained statistically significant.

### 3.2 Normative data

Table 5 presents descriptive data for the *Parla-dino* test in the normative sample (*n* = 105). No significant gender differences were found in the *Parla-dino* static or dynamic scores (U = 1,314, *p* = 0.781; U = 1,331, *p* = 0.868). For this reason, normative data are presented for the entire group. Similarly, no significant differences were observed between groups based on maternal education (U = 982, *p* = 0.155; U = 973, *p* = 0.136) or paternal education (U = 1,182, *p* = 0.719; U = 1,203, *p* = 0.826), used as indicators of SES. The values in Table 5 offer a reference framework for interpreting individual performance among first-grade Italian children. Additionally, Table 6 illustrates the threshold values: 13 children (12%) scored at or below the 10th percentile on the static score, while 12 children (11%) scored at or below the 10th percentile on the dynamic score. We also computed descriptive statistics and percentiles for the Phonological Processing Test (Table 7): in our normative sample, 11% of children (*n* = 12) produced 17 or more phonological processes, corresponding to the 10th percentile threshold. At the classification level, convergence emerged between the two instruments: 10 children who scored at or below the 10th percentile on the *Parla-dino* score also fell at or below the 10th percentile on the Phonological Processing Test.

**Table 5:** Means, standard deviations, and medians for the *Parla-dino* test in the normative sample of Italian first-grade students (*n* = 105).

***Parla-dino***	**Participants (** ***n** = **105*** **)**			
	***M***	***SD***	**Range**	**Median (50th)**
Static score (accuracy)	10.2	3.01	2–15	10
Dynamic score (accuracy)	49.4	8.06	28–60	51
Phonological process (errors)	7.62	5.43	0–24	6

**Table 6:** Threshold values for *Parla-dino* test (*n* = 105).

***Parla-dino***	**Participants (** ***n** = **105*** **)**				
	**10th**	**25th**	**50th**	**75th**	**90th**
Static score (accuracy)	6	8	10	12	14
Dynamic score (accuracy)	37	44	51	56	58
Phonological process (errors)	17	11	6	3	1

**Table 7:** Means, standard deviations, medians, and percentile values for the Phonological Processing Test (*n* = 105).

**Phonological processing test**	**Participants (** ***n** = **105*** **)**				
	***M***	***SD***	**Range**	**50th**	**10th**
Raw score (accuracy)	25.4	4.72	7–31	27	19
Phonological process (errors)	7.43	6.81	0–34	6	17

### 3.3 Sensitivity and specificity of the *Parla-dino* test

To assess the diagnostic utility of the *Parla-dino* test, we examined its sensitivity and specificity in this sample of 60 children, comparing performance between the TD and DLD groups. Based on the clinical cut-off at the 10th percentile (Table 6), children who obtained a dynamic score greater than 37 on the *Parla-dino* task were classified as having performance within the expected range; scores at or below this threshold indicated compromised performance. A contingency table analysis (Table 8) showed that 7 out of 15 children with DLD were correctly identified as having a deficit from *Parla-dino* test (sensitivity = 0.47); this percentage increased to 66% when considering the static score. Conversely, specificity was found to be 0.91, meaning that 91% of TD children were correctly identified as having an adequate performance.

**Table 8:** Contingency table of *Parla-dino* test performance by group status (DLD vs. TD).

**Group**	**Deficit (*n =* ≤ 37)**	**Normal range (*n =* >37)**	**Total (*n*)**
TD	4	41	45
DLD	7	8	15
Total	11	49	60

37 corresponds to the cut-off score for the dynamic score of the *Parla-dino* test.

We analyzed the linguistic profiles of 15 children with DLD to gain a deeper understanding of the test's sensitivity. Given that the *Parla-dino* test targets phonological processing skills, we investigated whether children within the DLD group exhibited specific deficits in this domain. Specifically, we focused on symptoms indicative of SSD, operationalized as high frequency of phonological processes. Among the 7 children with DLD who demonstrated compromised dynamic scores on *Parla-dino* test, all showed scores ≤ 10th percentile on the Phonological Processing Test (i.e., phonological process ≤ 10th percentile; see Table 7), which is widely used in Italy to identify phonological processing difficulties. In contrast, of the eight children with DLD who performed within the normative range on the *Parla-dino* test, all but one also scored above the 10th percentile on the Phonological Processing Test (Cossu, n.d.).

## 4 Discussion

In the present study, a new test was developed to evaluate a child's phonological processing skills. Italy currently needs clinical tools to detect and quantify phonological difficulties in children at the time of entry into primary school. This concerns both the assessment of children who have already been diagnosed with DLD and are undergoing intervention but who may still demonstrate residual phonological difficulties, and of children without a diagnosis, whose phonological system may nonetheless be fragile (Sansavini et al., 2021). This is because phonological processing difficulties that persist during the school years may affect both language and narrative development, as well as academic achievements. It is well established that impaired phonological processing plays a significant role in the literacy and math deficits experienced by students with Learning Disabilities (Bishop and Snowling, 2004; Catts et al., 2005; Snowling and Hulme, 2021). Their phonological processing difficulties are recognized as a critical factor in word-level and subword-level processing challenges, which are central to the difficulties in mapping spoken language to written language seen in both reading and spelling disorders (Bishop and Snowling, 2004; Catts et al., 2005). Furthermore, difficulties in phonological processing may negatively impact math learning (Durkin et al., 2015). Despite this evidence, the tools available in Italy to identify not yet fully automatized phonological processing skills in primary school children are limited or not always capable of detecting borderline profiles.

The new clinical tool introduced here, called *Parla-dino*, is based on the DA approach. This tool enables the observation of changes in the child's production through graduated prompts, integrating performance data with clinically relevant qualitative information. The sample for the *Parla-dino* test consisted of 105 monolingual, typically developing first-grade children. All participants attended Italian primary schools and shared homogeneous age and linguistic characteristics, making the sample broadly representative of the Italian first-grade population. A detailed description of convergent and discriminant validity evidence supporting the interpretation of the *Parla-dino* test scores was provided. The scores obtained by the children in the *Parla-dino* test strongly correlate (corrected for age) with those in Cossu's Phonological Processing Test (one of the most widely used tools in the Italian clinical field to evaluate phonological processing in schoolers), providing reasonable evidence that *Parla-dino* effectively measures phonological processing skills (convergent validity). The strong association observed between *Parla-dino* scores and the Phonological Processing Test is further supported by the classification pattern: most of the children identified at or below the 10th percentile by *Parla-dino* were likewise classified at or below the 10th percentile on the other task. This finding demonstrates that *Parla-dino* captures phonological processing difficulties in a manner highly consistent with a widely used clinical tool in the Italian context, thus providing additional evidence supporting its validity and representing a meaningful indicator of consistency between the two instruments. *Parla-dino* also proves capable of discriminating between a clinically identified population of children with DLD and TD peers. The performance on the *Parla-dino* test was significantly lower in the group of school-aged children with DLD (*n* = 15) compared to their TD matched peers (*n* = 45). This finding aligns with the results of (Dispaldro et al. 2013), which showed the same results in Italian preschoolers with specific language impairments. Logistic regression analysis further confirmed the discriminant validity of the tool. Specifically, children's performance on the *Parla-dino* test significantly predicted their group status (i.e., DLD vs. TD); in other words, as the *Parla-dino* dynamic score (i.e., number of correct words with graduated prompting) increased, the likelihood of being classified with DLD decreased. Furthermore, the average scores provide information indicating that the test is appropriate for this age group (it has neither ceiling nor floor effects), with scores ≤ the 10th percentile reflecting a clinical impairment (see CLASTA and FLI, 2019; Sansavini, 2021, p. 43).

*Parla-dino* also demonstrated excellent specificity, correctly identifying 91% of individuals without the disorder. However, its sensitivity appeared relatively low (47%), as it correctly classified 7 out of the 15 children with DLD as having impaired performance. These seven children also exhibited phonological processing difficulties on the Phonological Processing Test, with a frequency of phonological processes falling below the 10th percentile. In contrast, the remaining 8 children with DLD (53%), except for one child, scored within the normal range on both the *Parla-dino* and the Phonological Processing Test. The relatively low sensitivity observed for *Parla-dino* is likely related to the heterogeneity of the DLD population. This pattern suggests that *Parla-dino* may be particularly sensitive to detecting children with SSD or marked phonological and speech-related difficulties, which aligns with the intended purpose of the tool. Importantly, *Parla-dino* appears to capture phonological difficulties specifically, rather than the broader linguistic impairments that may characterize other DLD profiles. In this sense, its high specificity indicates that *Parla-dino* is especially reliable for ruling out children who do not present phonological deficits, although it identifies primarily those with pronounced speech difficulties. Given this profile, *Parla-dino* should not be used as a standalone tool. Rather, it may serve as a highly informative component of a comprehensive clinical assessment, particularly when integrated with instruments that evaluate the full range of language abilities beyond speech. Although the preliminary nature of this analysis and the small size of the DLD sample limit the generalizability of the findings, two observations emerge. First, several children with DLD continue to display phonological weaknesses in the first year of primary school. Second, the *Parla-dino* the Parla-dino task may offer a useful indication of phonological processing difficulties in school-aged children. Considering these findings, and in comparison with the assessment tools currently available for phonological skills in Italy, the *Parla-dino* task introduces key innovative features, most notably its DA framework. The DA supports clinical assessment by helping determine the child's current level of phonological processing and ZPD (Vygotsky, 1978), and by distinguishing between phonological and programming difficulties, driven by increased sequential demands (e.g., increased articulatory demands, length of the articulatory plan). *Parla-dino* allows clinicians to observe modifiability through the progressive morphological simplification of words: the graduated prompts preserve the core phonotactic properties of the original word, while reducing the length and complexity of the articulatory plan. When a child produces errors even on short phonological segments, this pattern is likely indicative of a primary phonological deficit, which precedes phonological programming difficulties. Conversely, when errors emerge as the phonotactic string becomes longer, the difficulty is expected to be consistent with a programming deficit. In this sense, DA provides clinically relevant information that static tests cannot capture and offers meaningful guidance for clinical decision-making and interventions.

From our results, children with DLD and phonological processing impairments demonstrated limited modifiability in their spoken word production, even when facilitative cues were provided. Furthermore, comparing static and dynamic performance could provide important clinical insight. While a subset of children with DLD (47%) performed significantly worse than TD peers on both static and dynamic scores of the task, 5 children with DLD (33%) did not fall below the clinical threshold in either condition. This subgroup may represent alternative DLD profiles, such as those primarily characterized by semantic or morphosyntactic impairments rather than phonological ones. Alternatively, these children may have compensated for earlier phonological difficulties or presented minor clinical symptoms. Interestingly, 3 out of 15 children with DLD (20%) exhibited a discrepancy between their static and dynamic performance. Although they performed poorly in the static assessment, they demonstrated marked improvement when provided with graduated prompts. As suggested by (Ramus and Szenkovits 2008), the concept of a “phonological deficit” may encompass heterogeneous underlying mechanisms, including variable access to phonological representations rather than their permanent impairment. Notably, two of these three children were the only ones in the DLD group with multilingual backgrounds, suggesting that linguistic exposure may influence responsiveness to dynamic scaffolding. It is also possible that their modifiability pattern reflects the effects of speech therapy.

These clinical observations highlight the utility of the dynamic assessment approach in identifying learning potential rather than mere performance and underscore its relevance in differential diagnosis within the DLD spectrum, as well as in planning intervention. Consistent with clinical experience, children with phonological processing deficits appear not to benefit from the phonological simplifications provided by the examiner, suggesting that this limited modifiability could be a clinical marker of phonological processing impairments. These preliminary results are consistent with findings from the literature on English-and French-speaking children with language impairment (Camilleri and Law, 2007; Hasson et al., 2013; Kehoe and Matrat, 2024; Peña et al., 2014). Preschoolers with language difficulties performed significantly differently from TD peers on dynamic assessment measures (Camilleri and Law, 2007). They required more extensive prompting to identify target words in receptive vocabulary tasks and showed weaker performance in the post-teaching expressive component (Hasson et al., 2013). Moreover, as suggested by (Hasson et al. 2013), the qualitative analysis of children's performance on the DA may help to discriminate core language deficits from differences due to a bilingual language learning context in children exposed to multiple languages.

*Parla-dino* may therefore serve as a helpful tool not only for assessment purposes but also for guiding intervention, contributing to a more accurate orientation of speech therapy. This new tool helps the clinician define intervention goals within the ZPD. Although Parla-dino does not include explicit teaching or metacognitive strategies, the graduated prompting pattern can provide valuable diagnostic information about the child's level of phonological modifiability and the type and amount of support required to achieve accurate production. By understanding the nature of the phonological difficulty—for instance, determining whether it is primarily related to phonotactic complexity, to the co-articulation of all components that constitute the word, or, as often occurs, to a combination of both—targeted and gradual intervention planning can be implemented. In this way, the task is not intended to offer prescriptive therapeutic guidelines but rather to inform clinical reasoning, allowing clinicians to calibrate the degree and type of mediation used in subsequent therapy sessions. The DA process encourages therapists to navigate the ZPD systematically, gradually reducing support as the child gains independence and internalizes new information. The *Parla-dino* test can therefore also be helpful in the retest phase, after a period of treatment, to monitor changes in response accuracy (static score) but, more importantly, the evolution of the dynamic profile by observing whether the child requires fewer prompts to produce the same word, indicating greater linguistic autonomy and increased learning potential.

Despite its strengths, this study has some limitations. First, the group of children with DLD is small and heterogeneous; therefore, findings should be interpreted with caution. Replicating the analyses with a larger sample of children with DLD could enhance the reliability and validity of our findings. Second, a specific focus on children with SSD, phonological processing difficulties, and multilingual backgrounds might reveal important distinctions. Future studies could also gather detailed information about the speech-language therapy history of children, including the frequency, duration, and type of intervention, to better understand its impact on phonological modifiability. Third, there is a need for longitudinal studies to assess whether performance on the *Parla-dino* task predicts later academic skills, such as reading, writing, and mathematical abilities; this would further enhance the predictive validity of the tool. Among the directions for future research, it would be of interest to examine the relationship between performance on the *Parla-dino* test and extra-linguistic demands (e.g., working memory tasks). Previous literature has highlighted how extra-linguistic abilities may contribute to explaining the observed language difficulties (Collisson et al., 2015; Leonard et al., 2013; Marini et al., 2020; Pettenati et al., 2015). Such relationships may provide important insights for assessment and intervention. Moreover, it is necessary to extend the normative data to subsequent school grades to address the national gap in DA tools for phonological abilities in Italian. Future research should also explore the potential application of *Parla-dino* in preschool children to support even earlier identification of phonological vulnerabilities. In this context, a psycholinguistic adaptation or a selective reduction of items may be required, as some item might be too complex for younger children due to their phonotactic structure.

In conclusion, the identification of persistent phonological processing difficulties at the beginning of primary school could support the implementation of targeted interventions, potentially preventing or reducing future learning difficulties. The *Parla-dino* test appears to be a valid measure of phonological processing for first-grade students, and it successfully discriminated between children with DLD and their TD peers. To our knowledge, the *Parla-dino* test is also the only standardized dynamic assessment tool of phonological processing available for Italian children. The test materials are well constructed, making the *Parla-dino* test easy to administer, score, and interpret. Extensive evidence of validity for the interpretation of *Parla-dino* test was provided. Given the importance of phonological variables to the development of basic literacy and math skills, the *Parla-dino* test is likely to yield clinically relevant information, primarily through the comparison of children's performance in static and dynamic conditions. This test may be helpful for clinical professionals, as it showed potential to discriminate children at risk for language and learning difficulties, in order to facilitate the provision of intervention services, evaluate the effectiveness of intervention efforts, and provide a comprehensive profile of the phonological processing skills underlying language and learning outcomes.

## Data Availability

The datasets presented in this article are not readily available because contain sensitive information about children. Requests to access the datasets should be directed to Silvia Stefanelli, silvia.stefanelli@unirsm.sm.
